# Coordination of EZH2 and SOX2 specifies human neural fate decision

**DOI:** 10.1186/s13619-021-00092-6

**Published:** 2021-09-06

**Authors:** Yuan Zhao, Tianyu Wang, Yanqi Zhang, Liang Shi, Cong Zhang, Jingyuan Zhang, Jiao Yao, Qianyu Chen, Xiaofen Zhong, Yanxing Wei, Yongli Shan, Guangjin Pan

**Affiliations:** 1 CAS Key Laboratory of Regenerative Biology, Centre for Regenerative Medicine and Health, Hong Kong Institute of Science and Innovation, Hong Kong, Guangzhou Institutes of Biomedicine and Health, Chinese Academy of Sciences, Guangzhou, 510530 China; 2 University of Chinese Academy of Sciences, Beijing, 100049 China; 3 Guangdong Provincial Key Laboratory of Stem Cell and Regenerative Medicine, South China Institute for Stem Cell Biology and Regenerative Medicine, Guangzhou Institutes of Biomedicine and Health, Chinese Academy of Sciences, Guangzhou, 510530 China; 4 Institute for Stem Cell and Regeneration, Chinese Academy of Sciences, Beijing, 100101 China; 5 Biomedical Sciences College & Shandong Medicinal Biotechnology Centre, Shandong First Medical University & Shandong Academy of Medical Sciences, Ji’nan, 250062 Shandong China; 6 Nanfang Hospital, Southern Medical University, Guangzhou, 510515 China; 7 Bioland Laboratory (Guangzhou Regenerative Medicine and Health Guangdong Laboratory), Guangzhou, 510005 China

## Abstract

Polycomb repressive complexes (PRCs) are essential in mouse gastrulation and specify neural ectoderm in human embryonic stem cells (hESCs), but the underlying molecular basis remains unclear. Here in this study, by employing an array of different approaches, such as gene knock-out, RNA-seq, ChIP-seq, et al., we uncover that EZH2, an important PRC factor, specifies the normal neural fate decision through repressing the competing meso/endoderm program. EZH2^−/−^ hESCs show an aberrant re-activation of meso/endoderm genes during neural induction. At the molecular level, EZH2 represses meso/endoderm genes while SOX2 activates the neural genes to coordinately specify the normal neural fate. Moreover, EZH2 also supports the proliferation of human neural progenitor cells (NPCs) through repressing the aberrant expression of meso/endoderm program during culture. Together, our findings uncover the coordination of epigenetic regulators such as EZH2 and lineage factors like SOX2 in normal neural fate decision.

**Supplementary Information:**

The online version contains supplementary material available at 10.1186/s13619-021-00092-6.

## Background

During development, the coordination of epigenetic regulators and linage specific transcription factors (TFs) plays critical roles in normal lineage fate decisions (Hirabayashi and Gotoh 2010; Yao et al. 2016; Zhang et al. 2019). For instance, polycomb repressive complexes (PRCs) that modify chromatins are essential in lineage commitment during early embryonic development (Di Croce and Helin 2013; Gifford et al. 2013; Nichols and Smith 2012; Surface et al. 2010). Polycomb group proteins (PcG proteins) form two PRC complexes, PRC1 and PRC2. PRC2 contains core components, EZH1, EZH2, EED and SUZ12, and catalyzes histone H3 lysine 27 tri-methylation (H3K27me3) while PRC1 contains RING1A and RING1B, the E3 ubiquitin ligases, and mediates mono-ubiquitination of histone H2A at lysine 119 (H2AK119ub1) (Schuettengruber et al. 2017; Simon and Kingston 2009). Ablations of PRC2 core components (EZH2, EED and SUZ12) leads to severe defect in mouse gastrulation, thus demonstrating their essential role in early development (Morin-Kensicki et al. 2001; O'Carroll et al. 2001; Pasini et al. 2004). On the other hand, PRC2 and H3K27me3 are also important to maintain the pluripotency of embryonic stem cells (ESCs) through occupying and repressing critical lineage and developmental genes (Bernstein et al. 2006; Boyer et al. 2006; Margueron and Reinberg 2011; Pan et al. 2007). Interestingly, loss of function of PRC2 results in different phenotypes between mouse and human ESCs. Mouse ESCs (mESCs) deleted of PRC2 core components such as *Suz12, Eed or Ezh2* maintain the undifferentiated state while human ESCs (hESCs) without these components exit pluripotency and undergo spontaneous differentiation (Chamberlain et al. 2008; Pasini et al. 2007; Saha et al. 2013; Shan et al. 2017). hESCs with PRC2 deletions specifically differentiate toward meso/endoderm fate rather than the expected all three germ layer fate, because that the three germ layer genes might be de-repressed upon their deletion (Collinson et al. 2016; Shan et al. 2017). Interestingly, *EZH2*^−/−^ hESCs fail to generate the whole neural ectoderm in teratoma formation (Shan et al. 2017). These findings suggest that PRC2 specifically regulates the neural ectoderm lineage decision at early developmental stage (Collinson et al. 2016; O'Carroll et al. 2001; Obier et al. 2015; Pasini et al. 2007; Shan et al. 2017).

Indeed, PRCs and H3K27me3 have been shown to regulate neurogenesis at different development stages and levels. PRC2 was shown to regulate the transcriptional program in adult mouse striatal neurons (von Schimmelmann et al. 2016). On the other hand, JMJD3, the demethylase of H3K27me3 is required for neuronogenesis of adult brain (Park et al. 2014). We also reported that human NPCs with JMJD3 deficiency failed to become neurons due to the accumulation of repressive H3K27me3 (Shan et al. 2020). These findings demonstrate that PRCs and its mediated H3K27me3 play essential roles to regulate the neural fate decision. Theoretically, lineage fate decision is highly coordinated at all levels to ensure the generation of normal stem/progenitor cells and then the downstream related subtype cells (Hirabayashi and Gotoh 2010; Yao et al. 2016; Zhang et al. 2019). However, how precisely that PRCs and H3K27me3 specify the neural fate at early development remains unclear. In this study, we show that EZH2 and SOX2 coordinately specify normal neural fate decision in hESCs, during which EZH2 mainly act to repress the competing meso/endoderm program while SOX2 activates neural gene program. Our findings provide insights in understanding the mechanism that the coordination of epigenetic factors and lineage specific transcription factors to determine normal lineage fate.

## Results

### *EZH2*^−/−^ hESCs fail to generate subtype neuron/glia cells

**Fig. 1:**
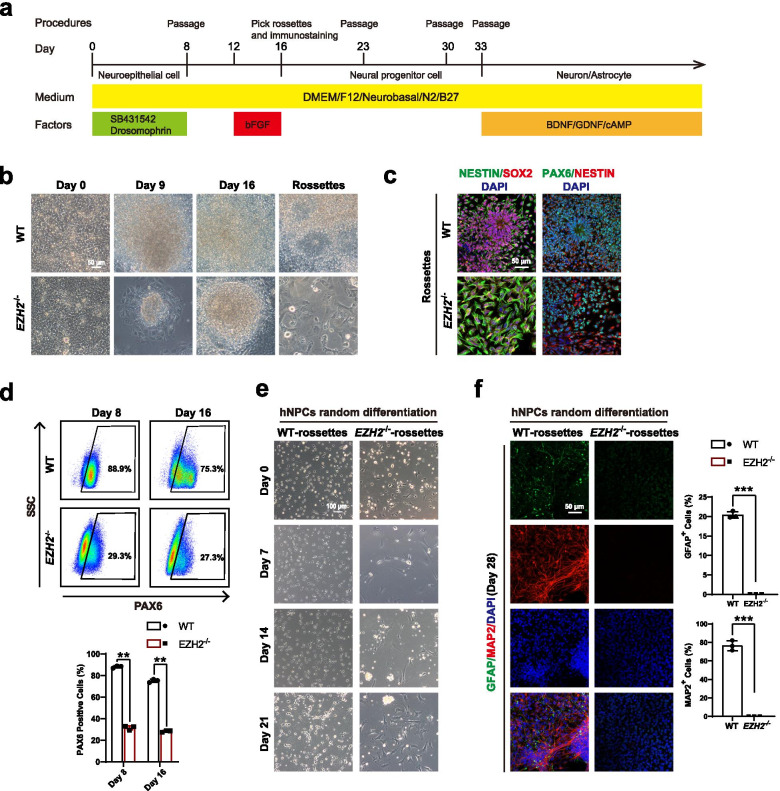
EZH2-deficient hESCs fail to differentiate into NPCs and subtype neuron/glia cells. **a** Schematic of the default neural differentiation and random differentiation strategy for human embryonic stem cells (hESCs) (more detail information in “Methods” sections). **b** Morphology of the wild-type (WT) H1 and *EZH2*^−/−^ hESCs during neural differentiation at day 0, day 9, day 16 and rosette-like cells. EZH2-deficient hESCs fail to form rosette-like cells. Scale bar, 50 μm. **c** Immunostaining for the NPC markers PAX6, SOX2, and NESTIN in WT rossettes and EZH2-mutant cells under neural differentiation. Scale bar, 50 μm. **d** FACS analysis of PAX6^+^ cells at day8 and day16 during neural differentiation in the indicated cells. The data represent the mean ± SD from three independent replicates (*n* = 3). Significance was determined using unpaired two-tailed Student’s t-tests. **, *P* < 0.01. **e** Morphology of the undifferentiated WT NPCs and EZH2-mutant NPCs and their differentiated cells at day 7, day 14 and day 28 during random differentiation. Scale bar, 100 μm. **f** Immunostaining for the marker of neuron MAP2 and the marker of astrocyte GFAP in the indicted differentiated cells at day 28 from NPCs. Scale bar, 50 μm. Quantity data from MAP2^+^ or GFAP^+^ cells were analyzed. Significance level was determined by unpaired two-tailed Student’s t-tests. ***, *P* < 0.001. The data represent the mean ± SD (standard deviation) from three independent replicates (*n* = 3). All error bars throughout the figure represent the SD (standard deviation) from three independent replicates (*n* = 3)

We have previously shown that *EZH2*^−/−^ hESCs failed to generate neural ectoderm in teratoma formation. To investigate the underlying molecular mechanism, we analyzed neural differentiation of *EZH2*-null hESCs under defined conditions in more detail (Fig. 1a). Upon dual SMADs inhibition (2i) to trigger neural differentiation, *EZH2*^−/−^ hESCs exited pluripotency but displayed a different morphology compared with wild type (WT) cells (Chambers et al. 2009) (Fig. 1b). In contrast to the morphology of WT neural cells, many flatten cells could be seen in *EZH2*^−/−^ hESCs differentiation (Fig. 1b). However, these *EZH2*^−/−^ hESCs derived cells triggered by neural condition did express neural fate related markers, such as PAX6, NESTIN, and SOX2 (Fig. 1c-d), albeit in reduced level compared with WT cells (Fig. 1d). We then performed downstream neuron/glia differentiation on WT and *EZH2*^−/−^ hESCs derived cells. While we could detect significant number of neuron and glia cells in neural differentiation of WT cells, but failed to detect these cells in *EZH2*^−/−^ hESCs derived cells based on morphology and immunostaining on specific markers, such as neuronal marker MAP2 and astrocyte marker GFAP (Fig. 1e-f). Together, these data demonstrate that *EZH2*^−/−^ hESCs derived neural cells are incompetent to generate downstream neural cells even though they expressed early NPC genes such as SOX2, PAX6 and NESTIN, etc.

### *EZH2*^−/−^ hESCs aberrantly express meso/endoderm genes during neural induction

**Fig. 2:**
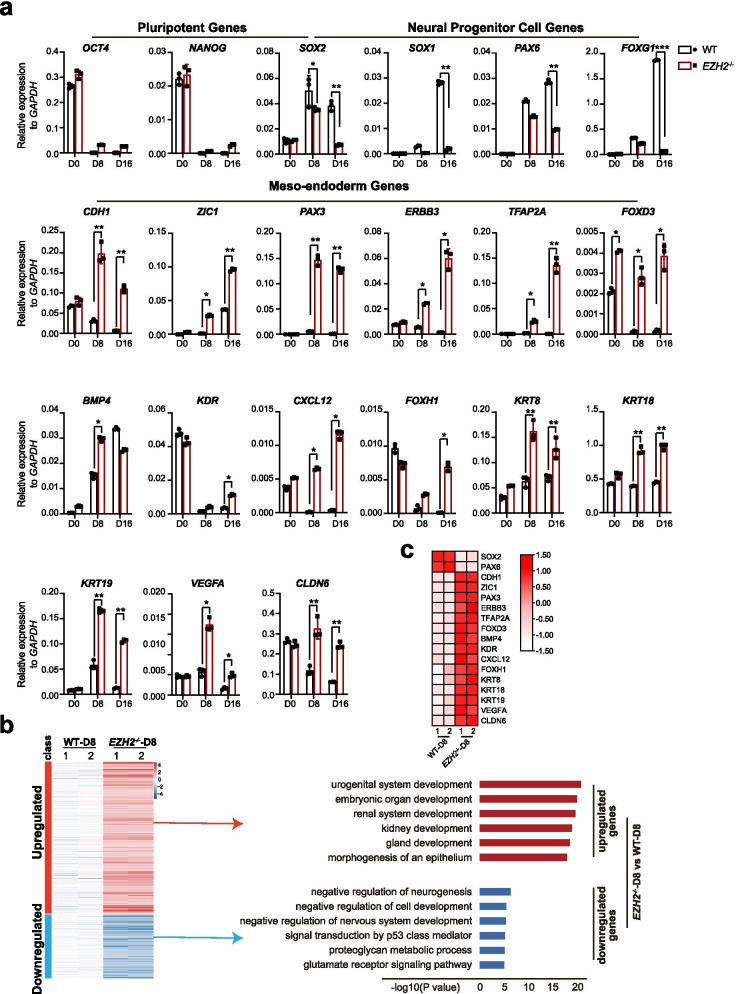
*EZH2*^−/−^ hESCs aberrantly express meso/endoderm genes during neural induction. **a** qRT-PCR analysis of the expression of the pluripotent genes *OCT4*/*NANOG*, the NPC genes *SOX2*/*SOX1*/*PAX6*/*FOXG1*, and meso-endoderm genes *CDH1*/*ZIC1*/*PAX3*/*ERBB3*/*TFAP2A*/ *FOXD3*/*BMP4*/*KDR*/*CXCL12*/*FOXH1*/*KRT8*/*KRT18*/*KRT19*/*VEGFA*/*CLDN6* at day 0, day 8 and day 16 during neural differentiation. Wild-type H1 hESCs served as controls. The data represent the mean ± SD (standard deviation) from three independent replicates (*n* = 3). Significance was determined using unpaired two-tailed Student’s t-tests. **, *P* < 0.01. *, *P* < 0.05. **b** Left panel, heatmap of up- or downregulated genes in EZH2-mutant and wild-type cells at day 8 of neural differentiation (EZH2^−/−^-D8 and WT-D8, respectively). Right panel, GO analysis for up- or downregulated genes in EZH2-mutant cells at day 8 of neural differentiation. **c**. Heatmap analysis for genes mentioned in Fig. 2a

We then analyzed gene expression profile in time course during neural differentiation of *EZH2*^−/−^ hESCs. Pluripotent markers such as *OCT4* and *NANOG* were successfully downregulated in both WT and *EZH2*^−/−^ hESCs, which is consistent to their differentiated morphology (Fig. 2a). Many NPC markers, such as *SOX2*, *PAX6* and *FOXG1* were induced in *EZH2*^−/−^ hESCs at early stage but become suppressed at later stage of neural differentiation compared with WT cells (Fig. 2a). Strikingly, a panel of meso-endoderm genes, such as *CDH1*, *ZIC1*, *PAX3*, *ERBB3*, *TFAP2A*, et al., were significantly de-repressed in *EZH2*^−/−^ cells compared to WT cells during neural differentiation (Fig. 2a). These data indicate that loss of EZH2 leads an aberrant activation of meso-endoderm gene program during neural fate commitment. We also performed whole genome transcriptome analysis between *EZH2*^−/−^ and WT cell line at day 8 of differentiation (hereafter denoted *EZH2*^−/−^-D8 and WT-D8) (Fig. 2b). Consistent to the suppressive function of EZH2, EZH2 deficiency leads to much more the up-regulated genes than the down-regulated ones in *EZH2*^−/−^-D8 cells compared with WT-D8 (Fig. 2b, right panel). These upregulated genes are mainly enriched in functions related to meso-endoderm development, such as kidney and organ development, while the small number of downregulated genes are related to neural functions (Fig. 2b, right panel, Fig. 2c). Together, these data demonstrate that the meso-endoderm genes are de-repressed and aberrantly re-activated in neural differentiation in the absence of EZH2.

### EZH2 suppresses meso-endoderm genes in normal neural fate decision

**Fig. 3:**
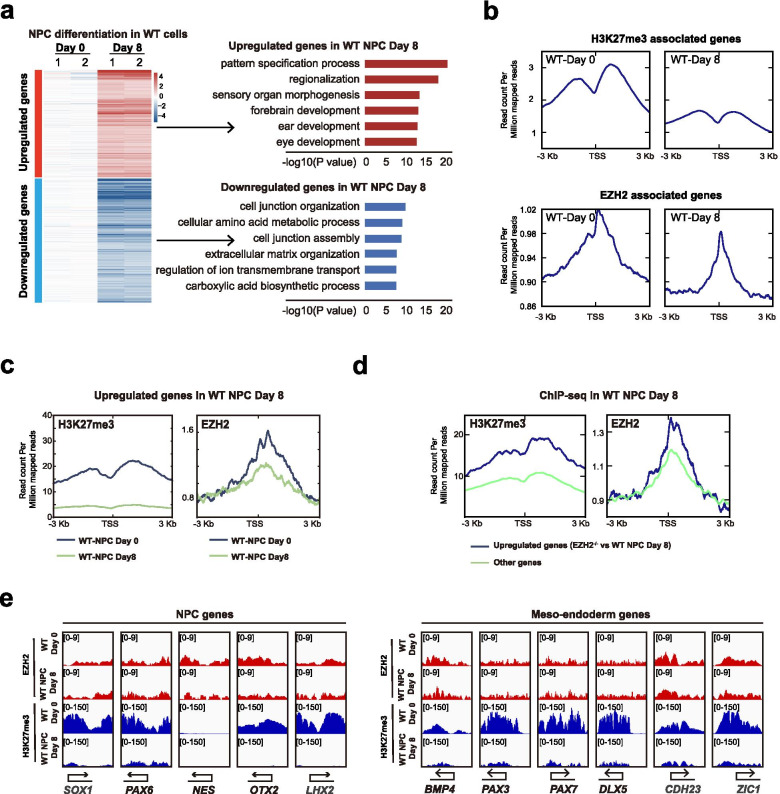
EZH2 suppresses meso-endoderm genes while releases neural genes in hESC neural differentiation. **a** Left panel, heatmap of up- or downregulated genes in wild-type cells at day 8 (WT Day 8) compared with WT cells at day 0 (WT Day 0) neural differentiation. Right panel, GO analysis for up- or downregulated genes in wild-type cells at day 8 (WT Day 8). **b** Signal densities of H3K27me3-associated genes and EZH2-associated genes in the indicated wild-type (WT) or EZH2-mutant cells at day 0 and day 8 of neural differentiation from ChIP-seq data. **c** Signal densities for H3K27me3 or EZH2 enrichment from wild-type cells at day 0 and day 8 ChIP-seq data for upregulated genes in wild-type cells at day 8 (WT Day 8). **d** Signal densities of H3K27me3 and EZH2 enrichment from WT-Day 8 ChIP-seq data for upregulated genes in EZH2-mutant cells at day 8 of neural differentiation. **e** Genomic views of the H3K27me3 modification and EZH2 enrichment for NPC genes and meso-endoderm genes in wild-type cells at day 0 and day 8 from ChIP-seq data

To further investigate the role of EZH2 in normal neural differentiation, we generated transcriptome data and ChIP-seq data of EZH2 and H3K27me3 in hESCs at day 0 and day 8 differentiation (WT-Day 0 or WT-Day 8, respectively) (Fig. 3a-b). As expected, the upregulated genes in WT-Day 8 cells were highly related to neuroectoderm functions (Fig. 3a). The downregulated genes were enriched in cell junction and extracellular matrix organization functions (Fig. 3a). For those up-regulated genes with neural functions, EZH2 binding as well as H3K27me3 binding were significantly reduced in WT-Day 8 cells compared with WT-Day 0 cells (Fig. 3b-c), demonstrating the neural genes lose EZH2 and H3K27me3 enrichment in neural differentiation. We also examined the EZH2 and H3K27me3 on those aberrantly expressed meso-endoderm genes due to loss of function of *EHZ2* (up-regulated genes in *EZH2*^−/−^-Day 8 VS WT-Day 8) (Fig. 3d). These EZH2 deficiency induced genes showed higher EZH2 binding and H3K27me3 compared with the randomly picked genes in WT-Day 8 (Fig. 3d). Consistently, many known critical meso-endoderm regulators such as *BMP4*, *PAX3*, *PAX7*, *DLX5*, etc. showed high enrichment of H3K27me3 and EZH2 in WT-Day8 cells while the essential neural genes lost H3K27me3 and EZH2 (Fig. 3e). Together, these data demonstrate that EZH2 acts to suppress meso/endoderm genes while release neural genes to specify neural fate in hESC differentiation.

### Coordination of EZH2 and SOX2 in neural lineage fate decision

**Fig. 4:**
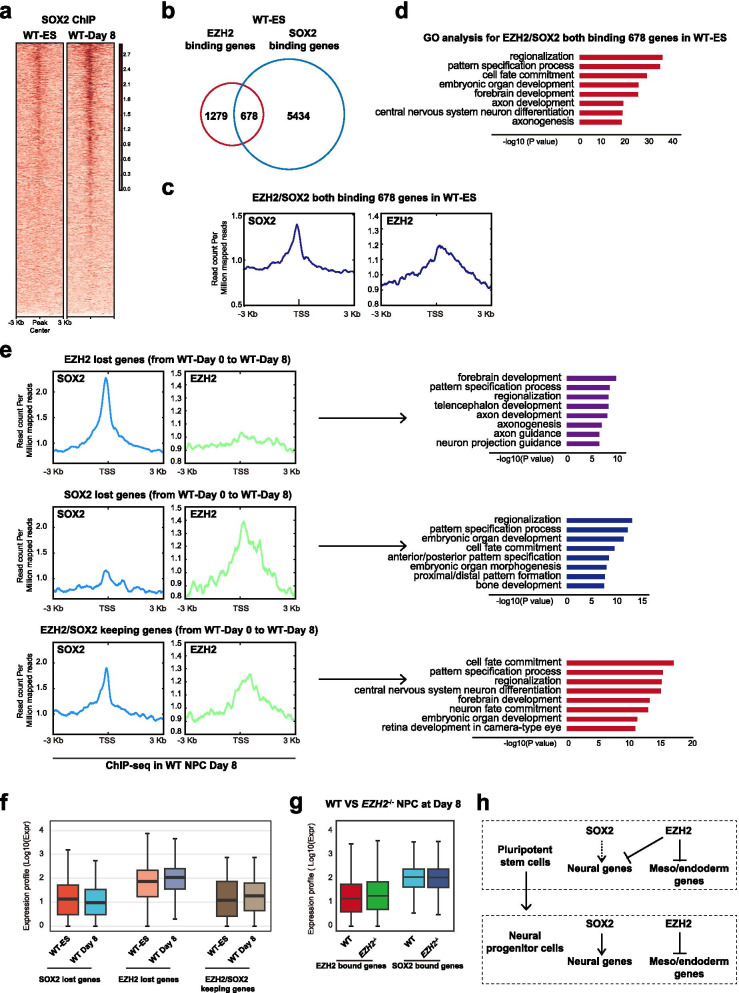
Coordination of EZH2 and SOX2 in neural lineage fate decision. **a** Signal densities heatmap of SOX2-ChIP-seq data indicating regions between WT ESCs and day 8 cells in neural differentiation (WT-Day 8). **b** Venn diagram for EZH2-binding genes and SOX2-binding genes in WT ESCs. **c** Signal densities of SOX2 and EZH2 enrichment for EZH2/SOX2 co-binding genes in WT ESCs. **d** GO analysis for EZH2/SOX2 both binding 678 genes in WT ESCs. **e** Left panel, Signal densities of SOX2 and EZH2 enrichment from WT-Day 8 ChIP-seq data for EZH2 lost genes, SOX2 lost genes or EZH2/SOX2 keeping genes in wild type cells at day 8 compared with day 0 in neural differentiation (WT-Day 8 and WT-Day 0, respectively). Right panel, GO analysis for EZH2 lost genes, SOX2 lost genes or EZH2/SOX2 keeping genes in WT-Day 8 cells. **f** RNA expression level in wild-type ESCs and day 8 cells in neural differentiation (WT-Day 8) for EZH2 lost genes, SOX2 lost genes or EZH2/SOX2 keeping genes. **g** RNA expression level in wild-type and EZH2^−/−^ cells at day 8 in neural differentiation for EZH2-bound and SOX2-bound genes, respectively. **h** The model for coordination of EZH2 and SOX2 in neural lineage fate decision from hESCs

SOX2 is the critical dual-role transcription factor that maintains pluripotency of ESCs and also regulates neural lineages (Zhou et al. 2016). To examine the coordination of EZH2 and SOX2 in neural fate decision, we performed ChIP-seq of SOX2 in neural differentiation of hESCs at Day 0 and Day 8. Generally, SOX2 intensity was much higher in Day 8 differentiated neural cells compared with Day 0 undifferentiated hESCs (Fig. 4a). Interestingly, about one third EZH2-bound promoters in undifferentiated WT hESCs were co-bound by SOX2 (Fig. 4b-c). These EZH2 /SOX2 co-bound genes were highly enriched in neural functions (Fig. 4c-d), indicating that the sub-set of neural genes suppressed by EZH2 are pre-bound by SOX2 in undifferentiated hESCs. We then tracked dynamic change of EZH2 and SOX2 binding on these genes in Day 8 neural differentiated cells (Fig. 4e). A portion of EZH2/SOX2 co-bound genes lost EZH2 but kept SOX2 in Day 8 neural cells and these genes were exclusively related to neural lineage functions (Fig. 4e upper panel). Another panel of genes lost SOX2 but kept EZH2 and they were related to A/P patterning as well as other non-neural fate functions such as bone development (Fig. 4e, middle panel). The third group genes kept both SOX2 and EZH2 and were related to functions of later stage neural development such as neuron maturation, retina development, etc. (Fig. 4e, lower panel). At the expression level, genes that lost SOX2 continue to be repressed in Day 8 differentiated cells, while those genes that lost EZH2 were de-repressed (Fig. 4f, left and middle panel). EZH2 /SOX2 keeping genes showed no big difference in expression between Day 8 differentiated cells and Day 0 undifferentiated hESCs (Fig. 4f, left and right panel). Upon *EZH2* deletion, EHZ2 bound genes were up-regulated in Day 8 differentiated cells while the SOX2 bound genes showed no big changes (Fig. 4g). In summary, these data reveal that in neural fate decision, SOX2 pre-binds critical neural fate genes that are directly suppressed by EZH2 in undifferentiated hESCs (Fig. 4h). These genes lose EZH2 and are up-regulated by SOX2 in neural fate cells (Fig. 4h). At the meanwhile, the competing meso/endoderm genes were solely bound and directly repressed by EZH2 to ensure the fidelity of neural fate (Fig. 4h). To confirm this model, we performed immuno-staining analysis for expression profiles of EZH2/SOX2 protein and found EZH2 and SOX2 were co-expressed in same cell population during neural differentiation of wild type hESCs at day 0 and day 8 (Supplementary Figure 1a). Furthermore, we performed knock-down of SOX2 in hESC neural differentiation (Supplementary Figure 1b). As expected, hESCs with SOX2 knock-down exited pluripotency normally during 2i induced neural differentiation, but the expression of neural linage genes was severely impacted (Supplementary Figure 1c-g). While, the meso/endoderm genes were not activated or changed upon SOX2 knock-down in neural differentiation (Supplementary Figure 1g). Together, these data demonstrate that SOX2 and EZH2 play different roles but act coordinately to specify neural fate decision in hESCs.

### EZH2 promotes the proliferation of human neural progenitor cells (NPCs) in vitro

**Fig. 5:**
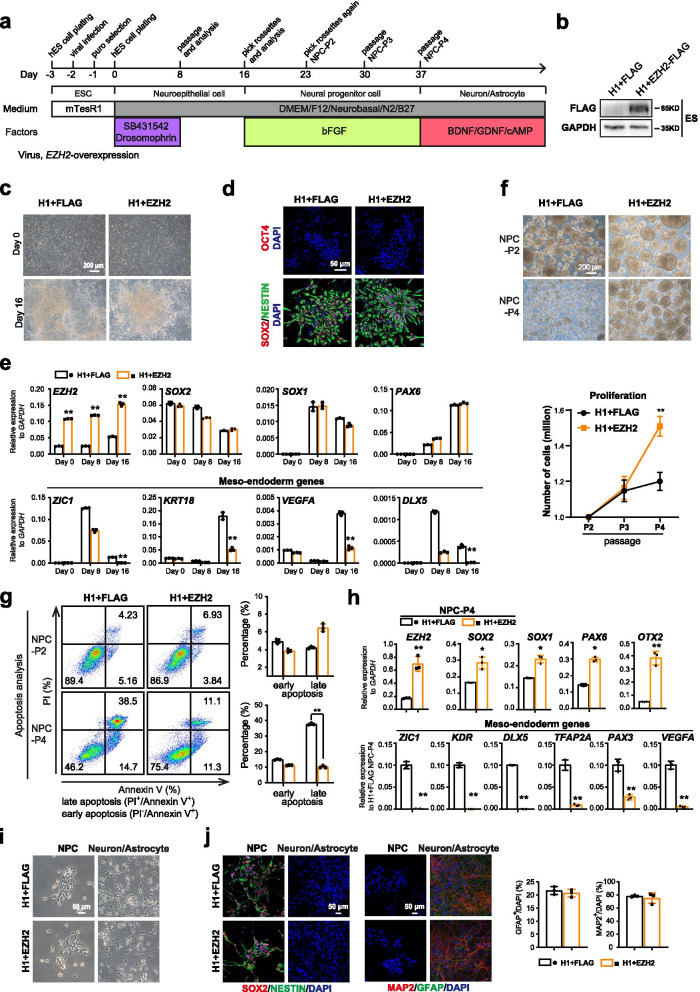
EZH2 promotes the proliferation of human neural progenitor cells (NPCs) in vitro*.* **a** Schematic of the default neural differentiation and random differentiation strategy for human embryonic stem cells (hESCs) with over-expression of EZH2. **b** Western blot for EZH2-FLAG proteins in the EZH2-overexpressed hES cells. **c** Morphology of the wild-type (WT) H1 and EZH2 over-expressed hESCs under NPC differentiation conditions at day 0 and day 16. Scale bar, 200 μm. **d** Immunostaining for the pluripotent marker OCT4 and the NPC markers SOX2/NESTIN in wild-type (WT) and EZH2 overexpressed rosette-like NPCs. Scale bar, 50 μm. **e** qRT-PCR analysis for the expression of *EZH2*, the NPC genes *SOX2*/*SOX1*/*PAX6*, and meso-endoderm genes *ZIC1*/*KRT18*/*VEGFA* /*DLX5* at day0, day8, day 16 of neural differentiation. Significance was determined using unpaired two-tailed Student’s t-tests. The data represent the mean ± SD (standard deviation) from three independent replicates (*n* = 3). **, *P* < 0.01. **f** Left panel, morphology of indicated NPCs maintained as neural spheres at passage 2 (P2) or passage 4 (P4). Scale bar, 200 μm. Right panel, proliferation curve of the indicated NPCs at different passages. Significance was determined using unpaired two-tailed Student’s t-tests. *, *P* < 0.05. **, *P* < 0.01. The data represent the mean ± SD (standard deviation) from three independent replicates (*n* = 3). **g** Apoptosis assay in the indicated cells at passage 2 (P2) or passage 4 (P4). PI- and/or annexin V-positive cells were analyzed by FACS. The significance level was determined using unpaired two-tailed Student’s t-tests. **, *P* < 0.01. Error bars represent the mean ± SD from three independent experiments (*n* = 3). **h** qRT-PCR analysis for the expression of *EZH2*, the NPC genes *SOX2*/*SOX1*/*PAX6*, and meso-endoderm genes *ZIC1*/*KDR*/*DLX5*/*TFAP2A* /*PAX3*/*VEGFA* in the indicated NPCs at passage 4 (P4). The significance level was determined using unpaired two-tailed Student’s t-tests. **, *P* < 0.01. Error bars represent the mean ± SD from three independent experiments (*n* = 3). **i** Morphology of NPCs and their subtype neuron/glia cells with over-expression of EZH2. Scale bar, 50 μm. **j** Immuno-staining analysis for SOX2/NES, MAP2/GFAP in NPCs with forced EZH2 expression during random differentiation. The numbers of MAP2^+^ or GFAP^+^ cells were analyzed. The significance level was determined using unpaired two-tailed Student’s t-tests. **, *P* < 0.01. Error bars represent the mean ± SD from three independent experiments (*n* = 3)

Since EZH2 plays critical roles in specifying neural fate through suppressing the competing meso-endoderm program, we then sought to examine whether EZH2 promote generation and proliferation of NPCs in vitro. We infected lentivirus expressing EZH2 into wild-type (WT) hESCs and performed neural differentiation (Fig. 5a-d). Consistently, the expressions of selected meso-endoderm genes were significantly suppressed in EZH2-expressing hESCs in neural differentiation (Fig. 5e). Furthermore, the generated NPCs with EZH2-expressing showed much better proliferation and lower apoptosis at higher passages (Fig. 5f-g). Interestingly, we found that the expressions of NPC genes were better while selected meso-endoderm genes were significantly repressed in EZH2-overexpressed NPCs at higher passages (Passage 4, P4) (Fig. 5h). Furthermore, hNPCs with overexpression of EZH2 could normally generate the subtype neuron/glia cells similar to wild type hNPCs during NPC random differentiation (Fig. 5i-j). This data indicate that forced expression of EZH2 do not affect differentiation of NPCs. Together, these data demonstrate that EZH2 promotes proliferation of human NPCs in vitro.

## Discussion

How precisely the epigenetic regulators and lineage TFs coordinate the cell fate decision remains elusive. PRC2 complex (its core component EZH2, EZH1, SUZ12 and EED) plays critical roles in early development as well as maintaining pluripotent stem cells (PSCs) (Gifford et al. 2013; Schuettengruber et al. 2017; Surface et al. 2010). The working model is that PRCs and their mediated H3K27me3 occupy and repress the developmental genes for three germ-layers in PSCs. These PRC-bound genes are considered to be in a repressive but poised state under the undifferentiated state of PSCs. However, human ESCs with PRC2 deficiency exit pluripotency and undergo spontaneous differentiation toward the meso-endoderm fate rather than the random three germ layer fate assumedly due to de-repression of these genes (Shan et al. 2017). Strikingly, EZH2^−/−^ hESCs failed to generate the whole ectoderm in teratoma formation, indicating EZH2 plays specific roles in ectoderm fate decision (Collinson et al. 2016; Shan et al. 2017). We then investigate the molecular basis underlying neural differentiation using EZH2^−/−^ hESCs as a model. At the molecular level, EZH2 mainly repress the competing meso-endoderm genes during neural fate decision while the neural lineage factor, SOX2 activates those neural related genes in this process. Therefore, the epigenetic regulator, EZH2 and lineage TF, SOX2 act in different aspect but coordinately specify the neural fate fidelity (Fig. 4e). Our findings provide a model and insight to understand the exact role of epigenetic regulators in specifying the lineage fate decision.

## Conclusions

This study demonstrates coordination of EZH2, an important PRC factor, and SOX2, an important neural transcript factor, in neural lineage fate decision. Human embryonic stem cells (hESCs) with *EZH2* deletion fail to generate neural progenitor cells (NPCs) and neural subtype neuron/glia cells because an aberrant re-activation of meso/endoderm genes during neural induction. Moreover, EZH2 represses meso/endoderm genes while SOX2 activates the neural genes in normal neural fate decision. Finally, the study shows that EZH2 and SOX2 coordinately specify the normal neural fate.

## Methods

### Cell culture

We used mTeSR1 (STEMCELL Technologies) to maintain the human embryonic stem cell line H1 (Wi Cell) and its knock-out cell line (H1-*EZH2*^−/−^) on Matrigel (Corning)-coated plates (Shan et al. 2017). These hES cells were passaged every 3 days, and the culture medium was changed with fresh culture medium every day. These cells were maintained at 5% CO_2_ and 37 °C.

### Neural progenitor cell (NPC) differentiation

Human ES cells with 100% cell confluence were seeded onto Matrigel-coated 6-well plates in mTeSR1 medium. After 1 day, the culture medium was changed with N2B27 medium (50% DMEM/F12 (Gibco), 50% neurobasal (Gibco), 0.5 × N2 (Gibco), 0.5 × B27 (Gibco), 1% Glutamax (Gibco), 1% NEAA (Gibco), 5 μg mL^−1^ insulin (Sigma), and 1 μg mL^−1^ heparin (Sigma)) with 5 μM SB431542 (Selleck) and 5 μM dorsomorphin (DM, Selleck). The medium was changed every day. After 8 days, the cells were passaged at a 1:2 ratio on new Matrigel-coated 6-well plates in N2B27 medium with 10 μM Y-27632 (Selleck). The medium was changed with fresh N2B27 medium every 2 days. For 12 to 16 days, the cells were cultured with N2B27 medium plus 20 ng mL^−1^ bFGF (PeproTech). After 16 days, the neural progenitor cells were picked. Then, these clones were seeded onto Matrigel-coated 6-well plates in N2B27 medium plus 10 μM Y-27632 and changed to N2B27 the next day. After 7 days, canonical neural rosettes were appeared. We picked these neural rosettes and named NPC passage 2 (NPC-P2). For proliferation assay, we digested NPC-P2 into single cells with Accutase (Sigma), then 1 × 10^6^ NPC cells were cultured in each low adhesion plate with N2B27 medium plus 10 μM Y-27632 and changed N2B27 medium next day. The medium was changed every 2 days. Every 7 days, these NPCs were passaged and counted. The following passaging NPCs were named NPC passage 3 (NPC-P3), passage 4 (NPC-P4) and so on. These NPCs were cultured in N2B27 medium, and the fresh medium was changed every 2 days.

### Random differentiation of human neural progenitor cells

Human neural progenitor cells at passage two (NPC-P2) were digested into single cells for random differentiation. 2 × 10^4^ neural progenitor cells were seeded onto 24-well plates coated with Matrigel in N2B27 medium plus 10 μM Y-27632. 1 day later, the culture medium was changed with N2B27 medium plus 20 ng mL^−1^ GDNF (PeproTech), 20 ng mL^−1^ BDNF (PeproTech), and 1 mM cAMP (Sigma). The medium was changed with the fresh medium every 2 days. After 28 days of differentiation, the canonical morphology of neuron and astrocyte was appeared, and the differentiated cells were examined by immuno-staining and qRT-PCR assay.

### Quantitative real-time PCR (qRT-PCR)

We performed the extraction of total RNA from cells with TRIzol (Invitrogen). And then, 2 μg total RNA was used for reverse-transcription with a HiScript III RT SuperMix for qPCR Kit (Vazyme). Then, we performed qRT-PCR assay with ChamQ SYBR qPCR Master Mix (Vazyme) and a CFX96 machine (Bio-Rad). We used GAPDH to normalize the qRT-PCR results for our samples. All data were analyzed with three replicates. All primer sequences are listed in Supplementary Table 1.

### Western blot analysis

To detect PAX6, SOX2 and EZH2-FLAG, the cells was lysed by RIPA buffer (Beyotime) on ice for 10 min. Whole-cell extracts were loaded to 12% SDS-PAGE with gel quickly preparation kit (Beyotime). Then, these samples from the SDS-PAGE gels were transferred to PVDF membranes (Millipore). Then, these PVDF membranes were incubated with the corresponding primary antibodies overnight at 4 °C. After washing three times with TBST for 10 min each time, these PVDF membranes were incubated with the corresponding HRP-conjugated secondary antibodies at room temperature (RT) for 2 h. After washing three times in TBST for 10 min each time, these PVDF membranes containing samples were detected by ECL (Millipore) and captured with a SmartChemi image analysis system (Sage Creation). The detailed information of the antibodies is listed in Supplementary Table 2.

### Flow cytometry analysis

The cells from these samples were digested into single cells with Accutase (Sigma). Then, these single cells were fixed in fixation buffer (BD Biosciences). After washing with PBS, these cells were permeabilized in perm/wash buffer (BD Biosciences) at 4 °C for 10 min. After washing, these cells were divided into two equal parts, one was incubated with the corresponding primary antibodies at 37 °C for 30 min, and another one was incubated with corresponding isotype control antibodies at 37 °C for 30 min. After washing, these sample cells were incubated with corresponding secondary antibodies at 37 °C for 30 min. After washing in PBS twice, these sample cells were resuspended in PBS. And then these cells were analyzed with a CytoFLEX S flow cytometer (Beckman). The detailed information of the antibodies is listed in Supplementary Table 2.

### Immuno-staining assay

Cells were seeded onto Matrigel-coated 24-well plates for immuno-staining assay. Then, the cells were fixed in 4% paraformaldehyde (PFA) for 20 min at room temperature (RT). After washing three times with PBS for 5 min each time, the cells were permeabilized and blocked with 0.3% Triton X-100 (Sigma), 10% goat serum in PBS. Meanwhile, the cells were incubated with corresponding primary antibodies overnight at 4 °C. After washing three times with PBS for 5 min each time, the cells were incubated with corresponding secondary antibodies for 1 h. Then, DAPI (Sigma) were added into incubation solution at room temperature for 5 min. After washing three times with PBS for 5 min each time, we placed the slide upside down on the microslide with fluorescence mounting medium (Dako). Images were captured with an LSM 800 microscope (Zeiss). The detailed information of the antibodies is listed in Supplementary Table 2.

### EdU assay and apoptosis analyses

We used a Click-iT™ EdU Alexa Fluor® 647 flow cytometry assay kit (Invitrogen) according to the manufacturer’s recommendations for EdU assay. 1 × 10^6^ wild-type NPCs and NPCs with overexpression of EZH2 were seeded onto Matrigel-coated 6-well plates in N2B27 medium with 10 μM EdU or without EdU for ~ 14 h. Then, we digested these cells into single cells, and the single cells were fixed in fixation buffer. After washing in PBS, the cells were permeabilized in perm/wash buffer at 4 °C for 10 min. After washing in PBS, the cells were incubated in PBS plus CuSO4, the Alexa Fluor® 647 azide, and Click-iT® EdU buffer additive at 37 °C for 30 min. After washing in PBS, the samples were analyzed with a CytoFLEX S flow cytometer.

We used an Annexin V-FITC/PI cell apoptosis detection kit (Vazyme) according to the manufacturer’s recommendations for Apoptosis analysis. We digested wild-type NPCs and NPCs with overexpression of EZH2 into single cells. And 1 × 10^5^ cells were incubated with binding buffer, Annexin V-FITC, and PI together at room temperature (RT) for 15 min. Then, these sample cells were analyzed with a CytoFLEX S flow cytometer.

### RNA-seq and heatmap analyses

We used TRIzol (MRC) to lyse wild-type and EZH2^−/−^ cells at Day 0 and Day 8 in NPC differentiation and extract total RNA according to the manufacturer’s recommendations. Then, we established sequencing libraries with a VAHTS mRNA-seq V3 Library Prep Kit for Illumina (Vazyme) according to the manufacturer’s recommendations. The sequencing libraries from these samples were run on a NextSeq system with a NextSeq 500 Mid Output kit (Illumina).

Then, we analyzed all RNA-seq data. Briefly, RNA-seq reads were filtered by Trimmomatic (v0.35) and then aligned to the human reference genome (hg38) using Hisat2 (v2.0.4) with default parameters. The sorted BAM files were used as inputs for HTSeq-count (v0.6) to obtain read counts per gene. R package ChIPpeakAnno (v3.16.1) was used to identify nearby genes from the peaks obtained from MACS and SICER. Gene Ontology (GO) analysis was performed using clusterProfiler (v3.18). EDASeq (v2.24) was used to perform normalization of RNA-seq read counts and differential expression analysis was undertaken using the DESeq2 (v1.30).

### ChIP-seq

We performed ChIP-seq for analyzing whole-genome binding and enrichment of EZH2, H3K27me3 and SOX2. Briefly, we used 1% formaldehyde (Sigma) in PBS to crosslink 1 × 10^7^ cells from each sample with rotation at room temperature for 10 min. Then, we added 0.125 M glycine to stop these crosslinking reactions with rotation at room temperature for 5 min. After washing in cold PBS twice, the cells were lysed with 1% SDS lysis buffer plus 1 mM PMSF and protease inhibitor cocktail, and the cells were sonicated to obtain 200–500 bp chromatin fragments. Then, ChIP dilution buffer was used to dilute these sonicated samples. The sonicated fragments from these samples were co-incubated with magnetic beads (Dynabeads protein A and G (1:1)) (Invitrogen) and 5 μg corresponding specific antibody (anti-H3K27me3 antibody (Diagenode, C15410069), anti-EZH2 antibody (CST, 5246) or anti-SOX2 antibody (R&D, AF2018), respectively) with rotation overnight at 4 °C. These magnetic beads/antibody-bound samples were washed with rotation at 4 °C for 5 min with the following series buffers: low-salt wash buffer (0.1% SDS, 1% Triton X-100, 2 mM EDTA, 20 mM Tris–HCl (pH 8.0), 150 mM NaCl), high-salt wash buffer(0.1% SDS, 1% Triton X-100, 2 mM EDTA, 20 mM Tris–HCl (pH 8.0), 500 mM NaCl), LiCl wash buffer (0.25 M LiCl, 1% IGEPAL-CA630 (Sigma), 1% deoxycholic acid (sodium salt), 1 mM EDTA, 10 mM Tris–HCl (pH 8.0)), and TE buffer (10 mM Tris–HCl (pH 8.0), 1 mM EDTA). Then, these magnetic beads/antibody-bound samples were eluted with Elution Buffer (1% SDS, 0.1 M NaHCO_3_). These eluted samples were reverse-crosslinked with 5 M NaCl overnight at 65 °C, and added RNase A (20 mg/mL) at 37 °C for 1–2 h, 1 M Tris–HCl (PH 6.5), 0.5 M EDTA, Proteinase K at 45 °C for 2 h. Then, we purified the ChIPed DNA from these samples for ChIP-Seq. We used a Qubit fluorometer (Invitrogen) to measure ChIPed DNA and corresponding input DNA, and generated DNA libraries with these DNA using a VAHTS Universal DNA Library Prep Kit for Illumina (Vazyme), respectively. We measured these DNA libraries with a Qubit fluorometer, and sequenced these DNA libraries on a NextSeq 500 platform.

ChIP-seq reads were were filtered by Trimmomatic (v0.35) and then aligned to the human reference genome (hg38) using Bowtie2 (v2.2.5) (Langmead and Salzberg 2012) with default parameters. PCR duplicate reads were removed using SAMtools (v1.3.1) (with the parameters "-F 1804 -f 2 -q 30") and Picard tools MarkDuplicates (v1.90). Peak calling was performed using the MACS2 (v2.1.0) callpeak module (with parameters "-p 0.01 –nomodel –extsize 150 -B –SPMR –keep-dup all") for transcription factor binding, and then only peaks with -log10(qvalue) more than 0.5 (EZH2) or 1 (SOX2) were keeped. Enriched regions for histone modification were called at the FDR level of 1% using SICER (v1.1) (Zang et al. 2009) with window sizes set to 200 bp, and gap sizes set to 600 bp (H3K27me3). Tracks of signal were computed using MACS2 bdgcmp module with parameter "-m FE". BigWig files were visualized using computeMatrix, plotHeatmap and plotProfile module in DeepTools (v2.4.2) (Ramirez et al. 2014). Gene Ontology (GO) analysis was performed using clusterProfiler (v3.18).

### Statistical analysis

In general, we present the results as the mean ± SD (standard deviation), and the results are calculated by Microsoft Excel and GraphPad Prism from at least three biological repeats. Unpaired two-tailed Student’s t-tests was used to determine the significance level between samples. Differences with a *P* value < 0.05 were considered significant differences. No samples were excluded for any analysis.

## Supplementary Information

**Additional file 1: Supplementary Figure 1.***SOX2 *knock-down reduces efficiency of neural differentiation. **a** Immuno-staining analysis for expression profiles of EZH2 and SOX2 during neural differentiation of hESCs at day 0 and day 8. **b** Schematic of the default neural differentiation strategy for hESCs after viral infection. hESCs were infected with *SOX2*-shRNAs. We used puromycin to select the cells with *SOX2*-shRNAs at day -1. **c.** Western blot of PAX6 and SOX2 proteins in the indicated cells at day 0 or day 16 of neural differentiation. **d.** FACS analysis of PAX6^+^ cells at day16 of neural differentiation in the indicated cells. The data represent the mean ± SD from three independent replicates (*n*=3). The significance level was determined using unpaired two-tailed Student’s t-tests. **, *P*< 0.01. **e.** Immunostaining for the pluripotent marker OCT4, the NPC markers SOX2/NESTIN in neural differentiation of WT and *SOX2*-shRNA cells. Scale bar, 50 μm. **f.** qRT-PCR analysis of the expression of the pluripotent genes OCT4/NANOG and SOX2 at day 0, day 8 and day 16 of neural differentiation. The data represent the mean ± SD (standard deviation) from three independent replicates (*n*=3). The significance level was determined using unpaired two-tailed Student’s t-tests. *, *P*< 0.05. **, *P*< 0.01. **g.** qRT-PCR analysis of the expression of the NPC genes *PAX6*/*SOX1*/*OTX2*/*FOXG1* and meso-endoderm genes *ZIC1*/*PAX3*/*KRT18*/*VEGFA* at day 16 of neural differentiation. The data represent the mean ± SD (standard deviation) from three independent replicates (*n*=3). The significance level was determined using unpaired two-tailed Student’s t-tests. **, *P*< 0.01

**Additional file 2: Supplementary Table 1** Primer sequences for this manuscript. **Supplementary Table 2** Antibody information for this manuscript.

## Data Availability

All data supporting the findings of this study are available within the article and its supplementary information files or from the corresponding upon reasonable request.
