# Supplementary figures and images for: Coordination of EZH2 and SOX2 specifies human neural fate decision
Source: Cell Regen. 2021 Sep 6;10:30. doi: 10.1186/s13619-021-00092-6 (PMC8421500; doi:10.1186/s13619-021-00092-6)

# Supplementary Figure 1

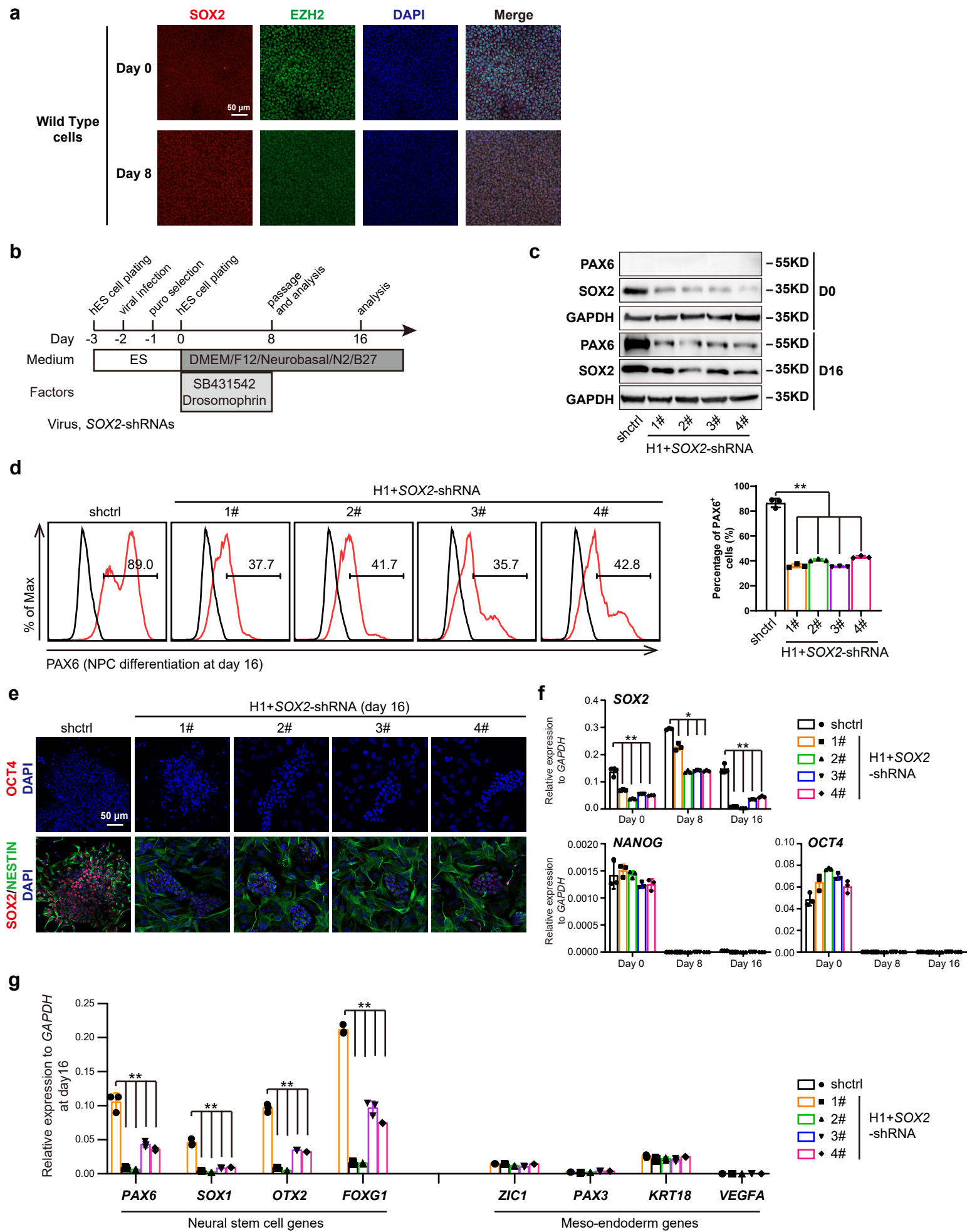

Supplement: Supplementary file 1 — Additional file 1: Supplementary Figure 1.SOX2 knock-down reduces efficiency of neural differentiation. a Immuno-staining analysis for expression profiles of EZH2 and SOX2 during neural differentiation of hESCs at day 0 and day 8. b Schematic of the default neural differentiation strategy for hESCs after viral infection. hESCs were infected with SOX2-shRNAs. We used puromycin to select the cells with SOX2-shRNAs at day -1. c. Western blot of PAX6 and SOX2 proteins in the indicated cells at day 0 or day 16 of neural differentiation. d. FACS analysis of PAX6+ cells at day16 of neural differentiation in the indicated cells. The data represent the mean ± SD from three independent replicates (n=3). The significance level was determined using unpaired two-tailed Student’s t-tests. **, P< 0.01. e. Immunostaining for the pluripotent marker OCT4, the NPC markers SOX2/NESTIN in neural differentiation of WT and SOX2-shRNA cells. Scale bar, 50 μm. f. qRT-PCR analysis of the expression of the pluripotent genes OCT4/NANOG and SOX2 at day 0, day 8 and day 16 of neural differentiation. The data represent the mean ± SD (standard deviation) from three independent replicates (n=3). The significance level was determined using unpaired two-tailed Student’s t-tests. *, P< 0.05. **, P< 0.01. g. qRT-PCR analysis of the expression of the NPC genes PAX6/SOX1/OTX2/FOXG1 and meso-endoderm genes ZIC1/PAX3/KRT18/VEGFA at day 16 of neural differentiation. The data represent the mean ± SD (standard deviation) from three independent replicates (n=3). The significance level was determined using unpaired two-tailed Student’s t-tests. **, P< 0.01 [file 13619_2021_92_MOESM1_ESM.pdf]
