# Supplementary material for: Coordination of EZH2 and SOX2 specifies human neural fate decision
Source: Cell Regen. 2021 Sep 6;10:30. doi: 10.1186/s13619-021-00092-6 (PMC8421500; doi:10.1186/s13619-021-00092-6)
Supplement: Supplementary file 2 — Additional file 2: Supplementary Table 1 Primer sequences for this manuscript. Supplementary Table 2 Antibody information for this manuscript. [file 13619_2021_92_MOESM2_ESM.pdf]

Supplementary Table 1 Primer sequences for this manuscript

Genes	Forward primer sequence	Reverse primer sequence
GAPDH	GGAGCGAGATCCCTCCAAAAT	GGCTGTTGTCATACTTCTCATGG
OCT4	CCTCACTTCACTGCACTGTA	CAGGTTTTCTTTCCCTAGCT
NANOG	TGAACCTCAGCTACAAACAG	TGGTGGTAGGAAGAGTAAAG
SOX2	CCCAGCAGACTTCACATGT	CCTCCCATTTCCCTCGTTTT
SOX1	AATTTTATTTTCGGCGTTGC	TGGGCTCTGTCTCTTAAATTTGT
PAX6	ATGTGTGAGTAAAATTCTGGGCA	GCTTACAACTTCTGGAGTCGCTA
FOXG1	GAGCGACGACGTGTTCATC	GCCGTTGTAACTCAAAGTGCTG
CDH1	GCTGGACCGAGAGAGTTTCC	CGACGTTAGCCTCGTTCTCA
ZIC1	ACATGAAGGTCCACGAATCCT	CTTGTGGTCGGGTTGTCTGT
PAX3	AGCTCGGCGGTGTTTTTATCA	CTGCACAGGATCTTGGAGACG
ERBB3	GGTGATGGGGAACCTTGAGAT	CTGTCACTTCTCGAATCCACTG
TFAP2A	GACTCGGAGACCTCTCGATCC	GACGGCATTGCTGTTGGAC
FOXD3	AGCCTAGTGAAGCCGCCTTACT	TTGTCGAACATGTCCTCGGACT
BMP4	CGTCCAAGCTATCTCGAGCC	CGGAATGGCTCCATAGGTCC
KDR	GAGGGGAACTGAAGACAGGC	GGCCAAGAGGCTTACCTAGC
CXCL12	GCCCTTCAGATTGTAGCCCG	GTAAGGGTTCCTCAGGCGTC
FOXH1	CCCCCAGAGGCAGAGTCG	GACCTGACGGATGATCTGGG
KRT8	CTCAAAGGCCAGAGGGCTTC	ACTTGGCGTTGGCATCCTTA
KRT18	GAGGGCTCAGATCTTCGCAA	CCAGCTGCAGTCGTGTGATA
KRT19	GTTCACCAGCCGGACTGAA	GCAGGTCAGTAACCTCGGAC
VEGFA	TCACCATGCAGATTATGCGGA	TACCGGGATTTCTTGCGCTT
CLDN6	TTATCTCCTTCGCAGTGCAGCTC	CACGATGCTGTTGCCGATGA
OTX2	CAAAGTGAGACCTGCCAAAAAGA	TGGACAAGGGATCTGACAGTG
EZH2	GCAGGCTGGGGGATTTTTATCA	AACGAATTTTGTTACCCTTGCGG
DLX5	CAGCCAAAGCTTATGCCGAC	CGGTCACTTCTTTCTCTGGCT
SOX2-shRNA1#	CAGCTCGCAGACCTACATGAA	
SOX2-shRNA2#	CGCTCATGAAGAAGGATAAGT	
SOX2-shRNA3#	GTACAGTATTTATCGAGATAA	
SOX2-shRNA4#	AGGAGCACCCGGATTATAAAT	

Supplementary Table 2 Antibody information for this manuscript

Name	Company	Cat. #	Host	Dilution
SOX2	R&D	mab2018	Mouse	1:1000
PAX6	Biolegend	PRB-278P	Rabbit	1:1000
FLAG	Sigma	F1804	Mouse	1:1000
HRP-GAPDH	Proteintech	HRP-60004	null	1:10000
PAX6-488	BD	561664	Mouse	1:100
NESTIN	Millipore	ABD69	Rabbit	1:1000
MAP2	CST	8707	Rabbit	1:1000
GFAP	Millipore	MAB360	Mouse	1:1000
OCT4	SantaCruz	SC-5279	Mouse	1:500
TUBB3	GeneTex	GTX130245	Rabbit	1:1000
EZH2	CST	5246S	Rabbit	1:100
H3K27me3	Diagenode	C15410069	Rabbit	1:100
SOX2	R&D	AF2018	Goat	1:100
